# Correction: Quantifying the spatiotemporal dynamics of the first two epidemic waves of SARS-CoV-2 infections in the United States

**DOI:** 10.1371/journal.pcbi.1014800

**Published:** 2026-09-15

**Authors:** Rafael Lopes, Yu Lan, Melanie H. Chitwood, Fayette Klaassen, Joshua A. Salomon, Nicolas A. Menzies, Joshua L. Warren, Nathan D. Grubaugh, Ted Cohen, Nicole A. Swartwood

In Fig 2, panel dates, y-axis label and scale are incorrect. Please see the correct Fig 2 here.

There are a number of errors in the legend of Fig 3. Please see the complete, correct Fig 3 legend here.

In the Spatiotemporal patterns of SARS-CoV-2 infections across the United States subsection of the Results, there are errors in the first paragraph. The correct paragraph is: As described in the Materials and Methods, we categorized infections as belonging to Wave 1 (September 17, 2020 to November 19, 2020) or Wave 2 (July 3, 2021 to September 4, 2021) (Fig 2A). Fig 2B – 2I shows the smoothed estimates of SARS-CoV-2 infections per 100,000 from the BYM2 model on eight dates, leading up to the peak of each of the two waves analyzed. Wave 1 originated with a set of hexes spanning central South Dakota, eastern North Dakota, and northeastern Montana (Fig 2B) in mid-September of 2020. Then the wave extended south and to both coasts, achieving a peak of 216 average daily infections per 100,000 persons by mid-November 2020 (Fig 2A and 2C – 2E). Wave 2 originated in the Ozarks (southern Missouri and northern Arkansas) (Fig 2F) in early July 2021 and then expanded further south (Fig 2G – 2I). Secondary epicenters of infection appeared later in July in the Pacific Northwest (Fig 2G), and the infection wave subsequently spread throughout the western United States (Fig 2G – 2I). This wave had a peak of 213 average daily infections per 100,000 persons by early September 2021.

In the Definition of a wave subsection of the Materials and methods, there is an error in the first sentence of the first paragraph. The correct sentence is: Using this definition, we categorized Wave 1 as the sets of contiguous hexagons above the threshold within the period ranging from September 17, 2020 to November 19, 2020, and Wave 2 as the sets of contiguous hexagons above the 190 infections per 100,000 threshold during the period from July 3, 2021 to September 4, 2021.

In the Wave expansion subsection of the Results, there are errors in the first paragraph. The correct paragraph is: Fig 3 shows contour plots of the speed of expansion for each wave. Wave 1 expanded from an area of 134,200 km2–6,515,300 km2 between September 17, 2020 to November 19, 2020 (Fig 3, ‘1st Wave’ bars). Wave 2 expanded from an area of 23,100 km2–7,573,500 km2 between July 3, 2021 and September 4, 2021 (Fig 3, ‘2nd Wave’ bars). The wave-like patterns were robust to the choice of threshold (S2 and S3 Figs).

There is an error in reference 56. The correct reference is: Freitas LP, Carabali M, Yuan M, Jaramillo-Ramirez GI, Balaguera CG, Restrepo BN, et al. Spatio-temporal clusters and patterns of spread of dengue, chikungunya, and Zika in Colombia. Ramos AN, editor. PLoS Negl Trop Dis [Internet]. 2022 Aug 23 [cited 2025 Mar 28];16(8):e0010334. Available from: https://dx.plos.org/10.1371/journal.pntd.0010334 5.

In the Data Availability statement, the Zenodo DOI is incorrect. The correct Zenodo DOI is: https://zenodo.org/records/19297984.

**Fig 2:**
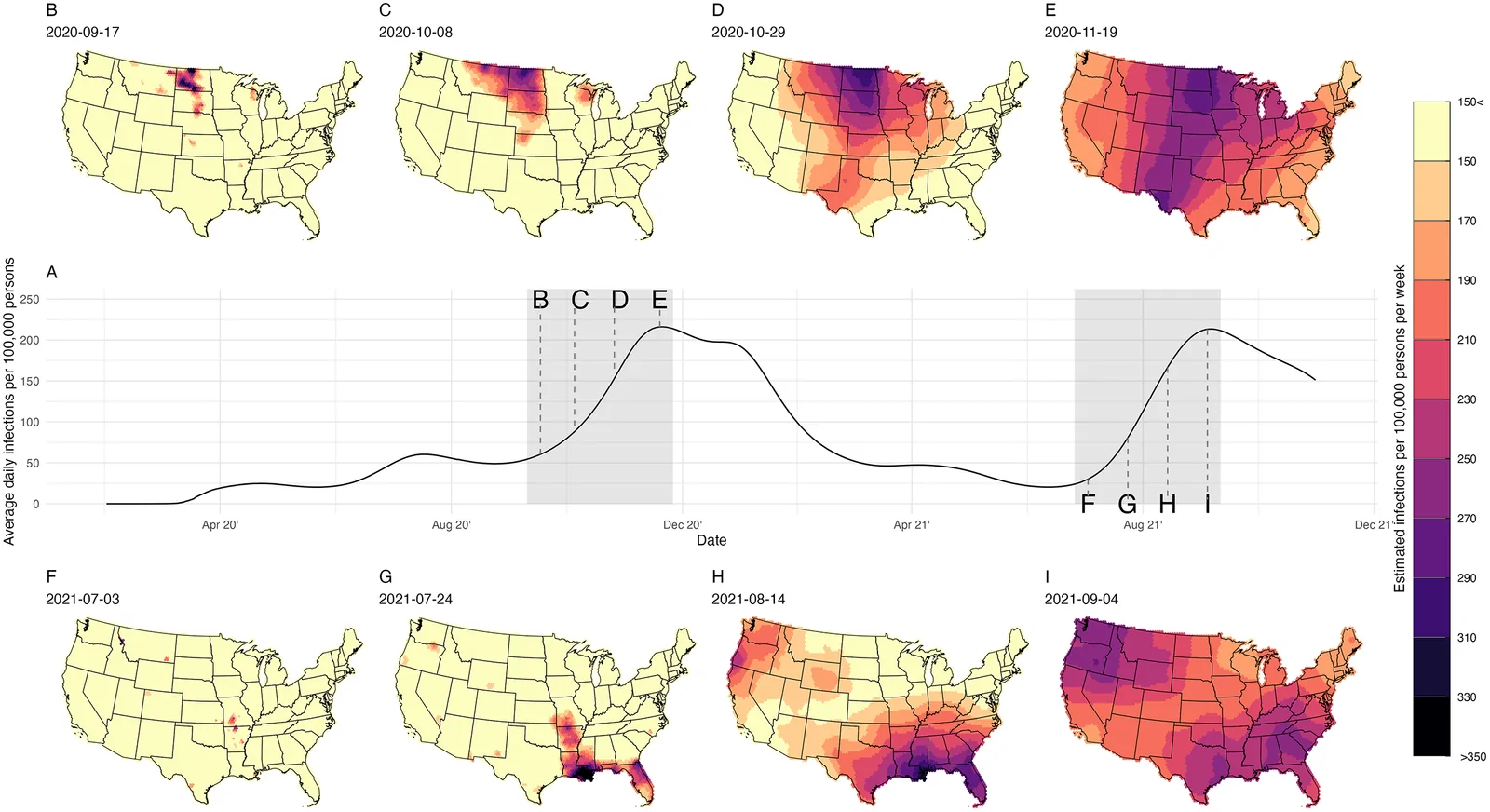
Estimated SARS-CoV-2 infections per 100,000 persons in the United States, March 2020–December 2021. Panel A: Time series of average SARS-CoV-2 infection per 100,000 persons for the United States; the gray shaded areas show the first two large waves of infections. Panels B, C, D, and E: Sequence of the spatially smoothed estimates of SARS-CoV-2 infections per 100,000 associated with Wave 1 at 4 time points. Panels F, G, H, and I: Sequence of the spatially smoothed estimates of SARS-CoV-2 infections per 100,000 associated with Wave 2 at 4 time points. All maps were generated using United States, state, and county borderlines maps in the public domain from the Census Bureau, which were downloaded through the R package Tigris [30]. The shapefile generated for this analysis with the population estimates and cumulative infection estimates can be found at: https://github.com/covidestim/waves/tree/waves-manuscript/Data/data-products. For a more detailed visualization of each wave, see the S1–S2 Movies.

**Fig 3:**
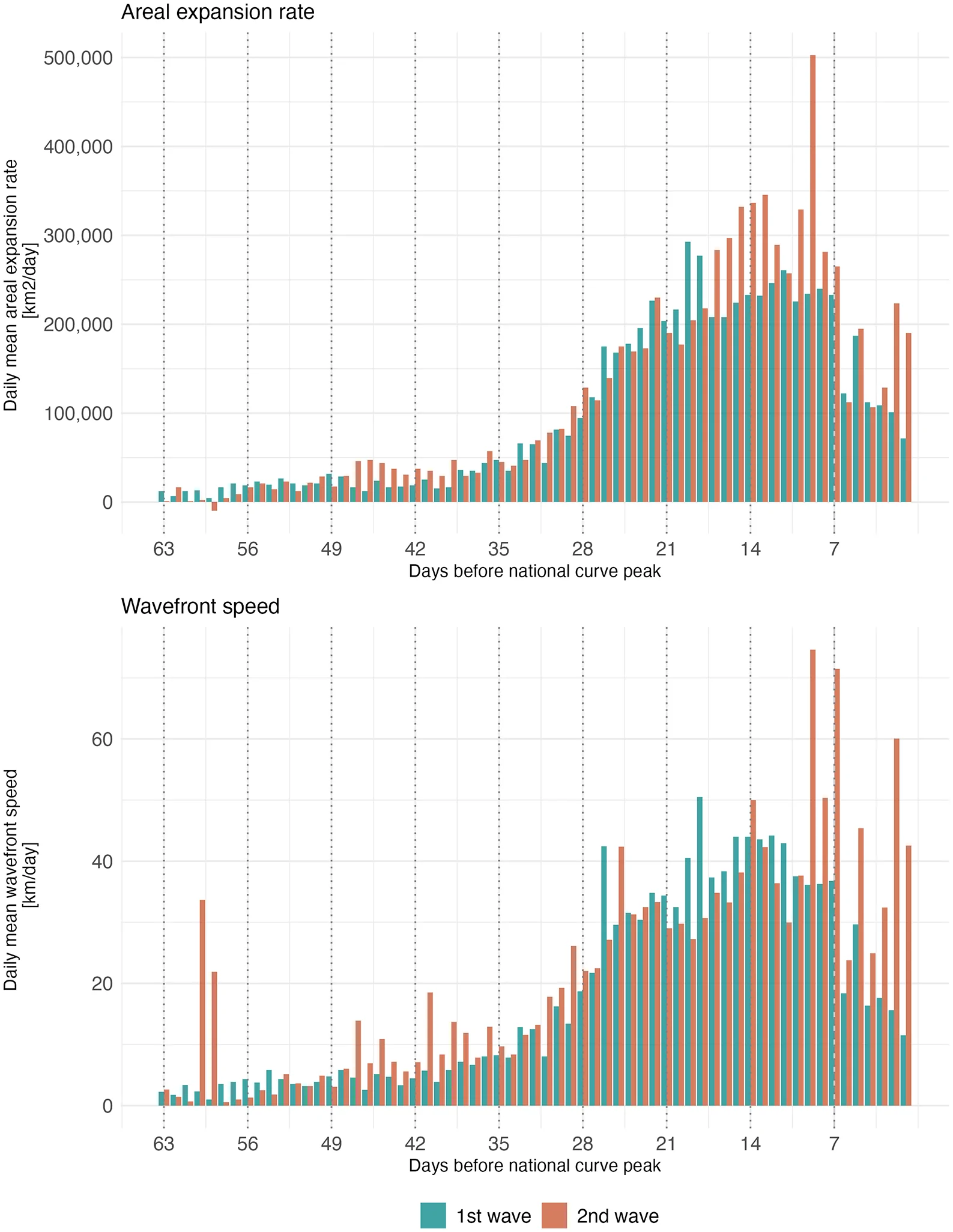
Daily mean areal expansion rate and wavefront speed for SARS-CoV-2 infection waves for 63 days preceding the infection per capita peak. (Wave 1: September 17, 2020–November 19, 2020; Wave 2: July 3, 2021–September 4, 2021). Top Panel: Mean daily areal expansion rate. Bottom Panel: Mean daily wavefront speed. Note: Areal expansion rate is the daily change in wave area. Wavefront speed is the distance from a vertex of the hexagonal grid at day d to the nearest point on the line at day d + 1.
