# Supplementary material for: Quantifying the spatiotemporal dynamics of the first two epidemic waves of SARS-CoV-2 infections in the United States
Source: PLoS Comput Biol. 2026 Mar 4;22(3):e1013983. doi: 10.1371/journal.pcbi.1013983 (PMC12959703; doi:10.1371/journal.pcbi.1013983)
Supplement: S1 Appendix — (DOCX) [file pcbi.1013983.s001.docx]

**Supplementary Material: Quantifying the spatiotemporal dynamics of the first two epidemic waves of SARS-CoV-2 infections in the United States**

**Short Title: Dynamics of the first two epidemic waves of SARS-CoV-2 infections in the US
Rafael Lopes^1,2,*^, Yu Lan^1,2^, Melanie H. Chitwood^1,2^, Fayette Klaassen^3^, Joshua A. Salomon^4^, Nicolas A. Menzies^3^, Joshua L. Warren^5,2^, Nathan D. Grubaugh^1,2^, Ted Cohen^1,2^, Nicole A. Swartwood^3, *, *,&^**

^1^ Department of Epidemiology of Microbial Diseases, Yale School of Public Health, Yale University, New Haven, Connecticut, United States of America

^2^ Public Health Modeling Unit, Yale School of Public Health, Yale University, New Haven, Connecticut, United States of America

^3^ Department of Global Health and Population, Harvard T.H. Chan School of Public Health, Harvard University, Boston, Massachusetts, United States of America

^4^ Department of Health Policy, Stanford University, Stanford, California, United States of America

^5^ Department of Biostatistics, Yale School of Public Health, Yale University, New Haven, Connecticut, United States of America

^&^Senior author

*[rafael.lopes@yale.edu](mailto:rafael.lopes@yale.edu) , [nswartwood@hsph.harvard.edu](mailto:nswartwood@hsph.harvard.edu)

**Prior choice for the modified Besag-York-Mollié model, BYM2**

We fitted the BYM2 model using R-INLA(1). The INLA methodology represents an approximation to full Markov chain Monte Carlo sampling algorithms, which can drastically reduce computational times, particularly for large analysis datasets. The original BYM model combines the Besag (7) version of an intrinsic conditional autoregressive (ICAR) model with an independent error term to balance spatial smoothness with non-spatial variability. BYM models use an adjacency matrix that defines regions as ‘neighbors’ based on whether they have touching boundaries. BYM2 is a reparametrized version of this model that leads to improvements when assigning prior distributions for model parameters and improved interpretations of those parameters (6). The BYM2 implementation in the R-INLA package makes use of penalized-complexity (PC) priors, which implies a risk decomposition as suggested by MacNab, 2011. From our model defined in (1) and following the reparameterization in Riebler et al., 2016 we have for $\theta(A_{i})$,

$\theta(A_{i}) = \frac{1}{\tau_{b}}(\sqrt{1-\phi}v(A_{i})+\sqrt{\phi}u(A_{i})$ (1)

where $\tau_{b}$ is a precision parameter and $\phi$ is the mixing parameter. Here, the $v(A_{i})$ are *iid* with a normal distribution and variance equal to one, and the $u(A_{i})$ parameters are assigned the Besag intrinsic conditional autoregressive (ICAR) model with variance equal to 1.

This reparameterization of the BYM model can be seen as a mixture between pure overdispersion (when $\phi= 0$), and a completely spatially structured risk (when $\phi= 1$ the Besag model). We used a conservative PC-prior for $\phi$, specifically, P($\phi>1/2=2/3)$ that assumes the unstructured random effect accounts for more variability than the spatially structured effect. For the precision parameter, $\tau_{b}$, we also used a PC-prior with parameterization P($\tau_{b} >0.2=0.01).$

**Sensitivity Analysis on infections per 100,000 surface threshold**

In the section **Definition of wave and speed of expansion of waves**, we define the threshold for a high rate of infection per 100,000 surfaces in the speed of invasion calculation as any value above the 75^th^ percentile (**S1B Fig**) of the surface values distribution, which corresponds to a value of 190 or more infections per 100,000. To test whether this threshold changes the assessment of per 100,000 infection surfaces and the wave-like patterns visualization, we conducted the following sensitivity analysis. First, we inspected the histogram of values of the infection rate surface for all the dates over all the hexes, **S1A Fig.** We found that most of the mass of the distribution, disregarding the values close to 0, is around 200 infections per 100,000, with a steep decrease to values greater than 233 infections per 100,000. To further check if the value of 190 infections per 100,000 is a reasonable choice for the threshold, we inspected the empirical cumulative density function (ECDF) of the denoised and spatially smoothed random effects (**S1B Fig**). Setting the threshold to a value equal to or greater than 190 infections per 100,000 which we think is the best choice. At much lower threshold values, deviation of values close to 0 will put more hexagons as being part of a wave. If the value of infections per 100,000 is greater than 190, we conclude from the shape of the ECDF function that it will be hard to classify any region as having a high rate of infection. Of course, the value can be set to bigger or smaller values than 190 infections per 100,000, but the wave-like pattern does not disappear. **S2 Fig** shows the speed of invasion for both waves, calculated with different thresholds, 127 or more infections per 100,000 for panel **A** and 233 infections per 100,000 for panel **B**. Those values are the median and the 90^th^ percentile of the distribution.

To check whether the wave-like pattern is sensitive to the choice of threshold, we recreated **Fig 2** for the same eight dates using a continuous scale (**S3 Fig)** or by setting the threshold to the median of the infections per 100,000 distribution (127 infections per 100,000) (**S4 Fig**).

**References:**

1. Lindgren F, Rue H. Bayesian Spatial Modelling with R-INLA. J Stat Softw [Internet]. 2015 Feb 16 [cited 2024 Oct 14];63:1–25. Available from: https://doi.org/10.18637/jss.v063.i19

2. MacNab YC. On Gaussian Markov random fields and Bayesian disease mapping. Stat Methods Med Res [Internet]. 2011 Feb 1 [cited 2024 Oct 10];20(1):49–68. Available from: https://doi.org/10.1177/0962280210371561

3. Riebler A, Sørbye SH, Simpson D, Rue H. An intuitive Bayesian spatial model for disease mapping that accounts for scaling [Internet]. arXiv; 2016 [cited 2024 Oct 2]. Available from: http://arxiv.org/abs/1601.01180
