# Supplementary figures and images for: Quantifying the spatiotemporal dynamics of the first two epidemic waves of SARS-CoV-2 infections in the United States
Source: PLoS Comput Biol. 2026 Mar 4;22(3):e1013983. doi: 10.1371/journal.pcbi.1013983 (PMC12959703; doi:10.1371/journal.pcbi.1013983)

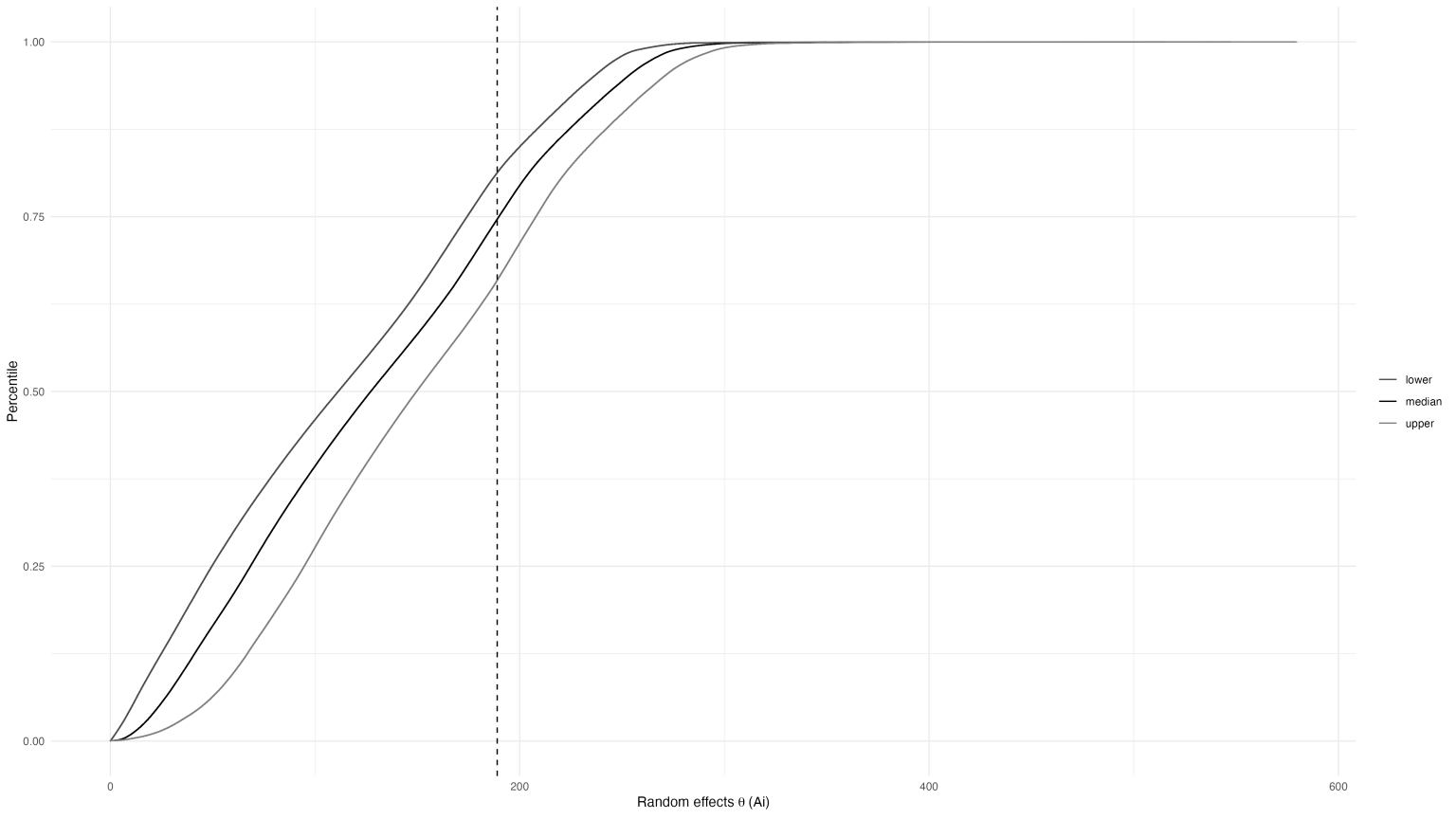

Supplement: S1 Fig — The ECDF shows that a threshold of 190 infections per 100,000 is indicated by the vertical dashed line at which the ECDF crosses the 75th percentile. The ECDF for the lower and upper bounds is shown in grey. (TIF) [file pcbi.1013983.s002.tif]

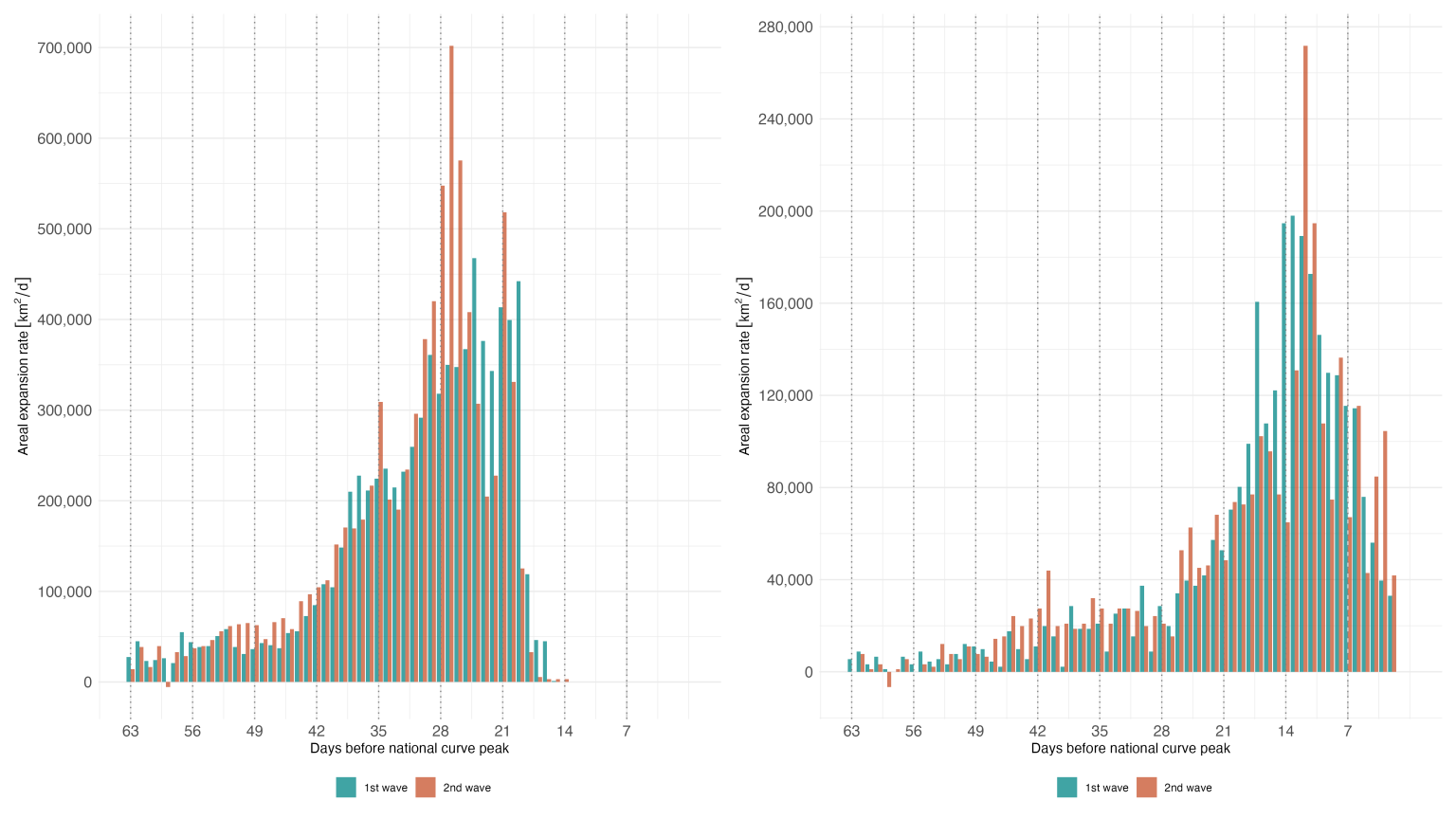

Supplement: S2 Fig — Areal wave expansion (km2/day) for different thresholds of infection per 100,00 in the progression calculation of the surfaces. Panel A is built with a threshold of 127 or more infections per 100,000; panel B is built with a threshold of 233 infections per 100,000. As in Fig 3C, we observe a maximal speed and a steep decrease after the peak, and the second wave had a higher invasion speed and encompassed a larger area at peak than the first wave. (TIF) [file pcbi.1013983.s003.tif]

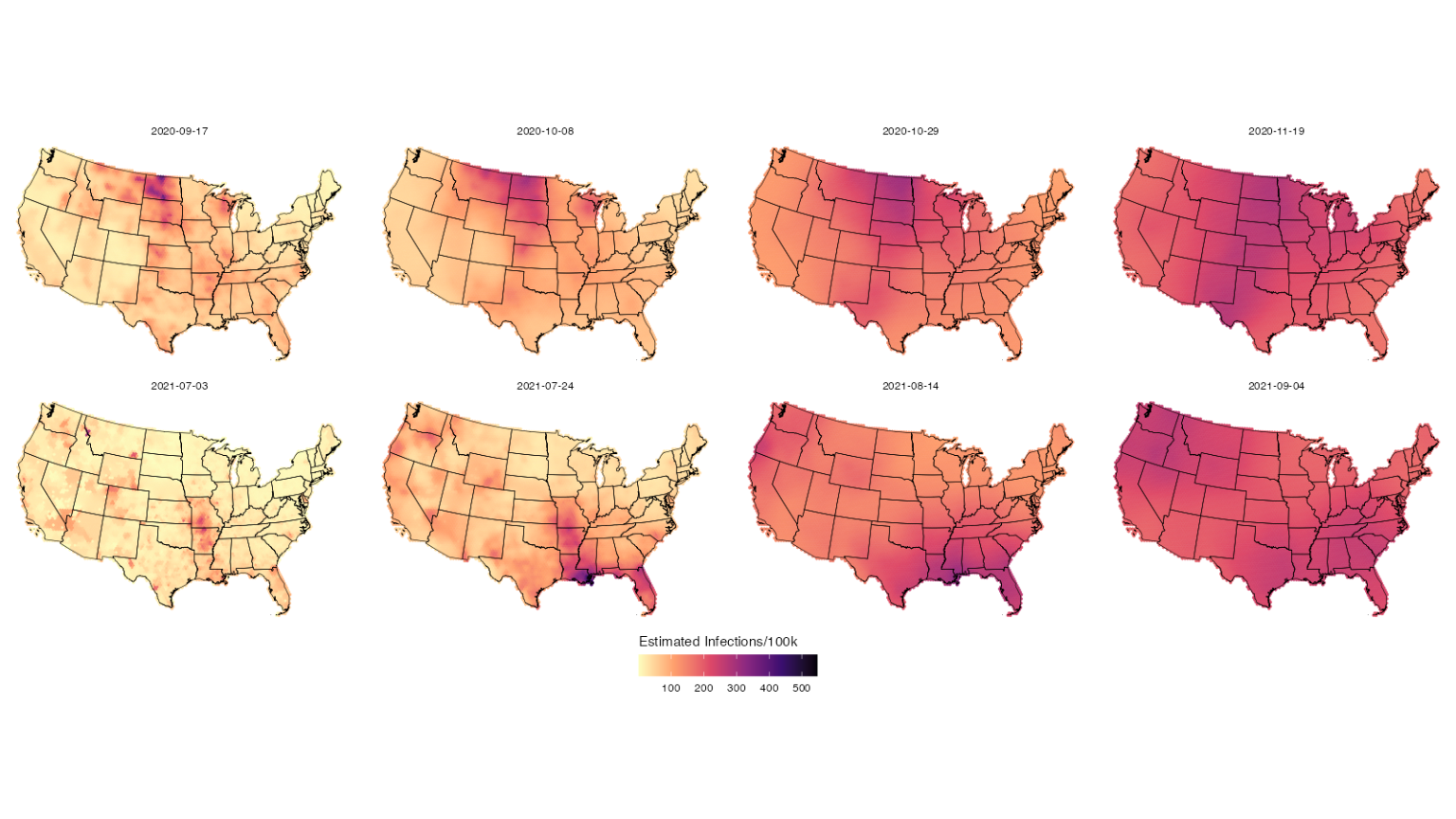

Supplement: S3 Fig — As expected, the wave-like pattern holds independently of the scale to be displayed, and as being an output of spatial smooth model, the continuous scale gives a less defined border to the risk surface expansion. All maps were generated using United States, state, and county borderlines maps in public domain from the Census Bureau which were downloaded through the R package Tigris [30]. The shapefile generated for this analysis with the population estimates and cumulative infections estimates can be found at: https://github.com/covidestim/waves/tree/waves-manuscript/Data/data-products. (TIF) [file pcbi.1013983.s004.tif]

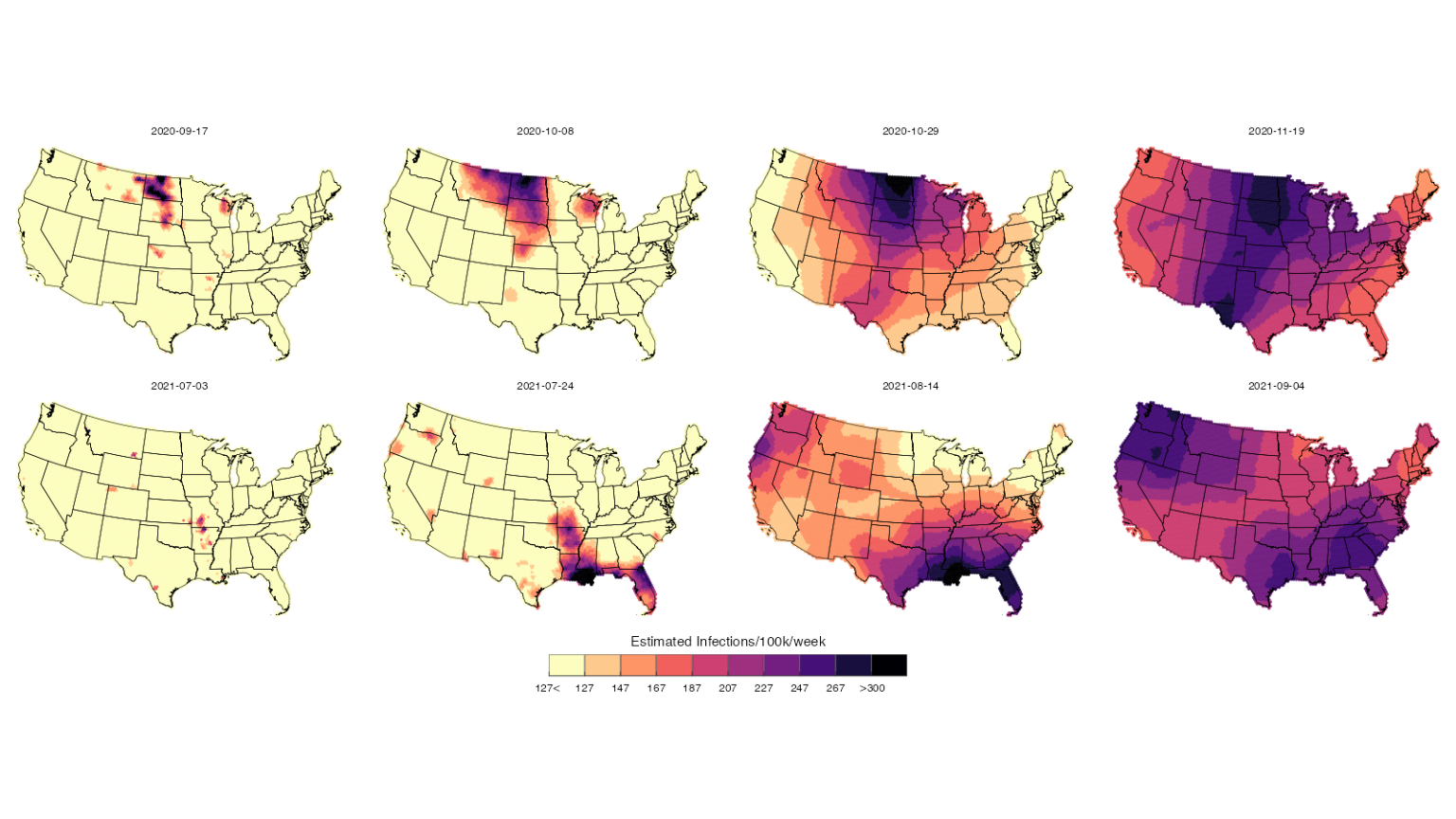

Supplement: S4 Fig — With a lower threshold showing on the map, the spread process seems to happen faster. All maps were generated using United States, state, and county borderlines maps in public domain from the Census Bureau which were downloaded through the R package Tigris [30]. The shapefile generated for this analysis with the population estimates and cumulative infections estimates can be found at: https://github.com/covidestim/waves/tree/waves-manuscript/Data/data-products. (TIF) [file pcbi.1013983.s005.tif]
