# Supplementary material for: Quantifying the spatiotemporal dynamics of the first two epidemic waves of SARS-CoV-2 infections in the United States
Source: PLoS Comput Biol. 2026 Mar 4;22(3):e1013983. doi: 10.1371/journal.pcbi.1013983 (PMC12959703; doi:10.1371/journal.pcbi.1013983)
Supplement: S1 Table — (DOCX) [file pcbi.1013983.s006.docx]

**S1 Table – Daily estimates of wave length, areal growth, and speed for each wave**

|  | Wave 1 | | | | | Wave 2 | | | | |
| --- | --- | --- | --- | --- | --- | --- | --- | --- | --- | --- |
| Days before peak | Wave edge length (km) | Areal wave expansion (km2/day) | Median speed (km/day) | Mean speed (km/day) | Length of wave edge with speed greater than or equal to overall median max speed | Wave edge length (km) | Areal wave expansion (km2/day) | Median speed (km/day) | Mean speed (km/day) | Length of wave edge with speed greater than or equal to overall median max speed |
| 63 | 3251.1 | 12100.0 | 0.0 | 2.3 | 535.0 | 1605.0 | 1100.0 | 0.0 | 2.6 | 164.6 |
| 62 | 3436.3 | 6600.0 | 0.0 | 1.8 | 391.0 | 1769.6 | 16500.0 | 0.0 | 1.4 | 205.8 |
| 61 | 3395.1 | 12100.0 | 0.0 | 3.4 | 637.9 | 2860.1 | 1100.0 | 0.0 | 0.7 | 123.5 |
| 60 | 3354.0 | 13200.0 | 0.0 | 2.3 | 535.0 | 2983.6 | 2200.0 | 0.0 | 33.6 | 370.4 |
| 59 | 3724.3 | 4400.0 | 0.0 | 1.0 | 246.9 | 3065.9 | -9900.0 | 0.0 | 21.9 | 946.5 |
| 58 | 3868.4 | 16500.0 | 0.0 | 3.5 | 864.2 | 2263.4 | 4400.0 | 0.0 | 0.6 | 102.9 |
| 57 | 3868.4 | 20900.0 | 0.0 | 3.9 | 905.4 | 2510.3 | 8800.0 | 0.0 | 1.0 | 185.2 |
| 56 | 4074.1 | 18700.0 | 0.0 | 4.3 | 967.1 | 2963.0 | 16500.0 | 0.0 | 1.3 | 288.1 |
| 55 | 3868.4 | 23100.0 | 0.0 | 3.8 | 864.2 | 3765.5 | 20900.0 | 0.0 | 2.5 | 720.2 |
| 54 | 4156.4 | 19800.0 | 0.0 | 5.8 | 967.1 | 4094.7 | 14300.0 | 0.0 | 1.8 | 555.6 |
| 53 | 4094.7 | 26400.0 | 0.0 | 4.3 | 1028.8 | 4341.6 | 23100.0 | 0.0 | 5.2 | 1028.8 |
| 52 | 4485.7 | 20900.0 | 0.0 | 3.5 | 987.7 | 4444.5 | 12100.0 | 0.0 | 3.6 | 658.4 |
| 51 | 4568.0 | 18700.0 | 0.0 | 3.2 | 884.8 | 4609.1 | 22000.0 | 0.0 | 3.2 | 946.5 |
| 50 | 4691.4 | 20900.0 | 0.0 | 3.9 | 987.7 | 4938.3 | 28600.0 | 0.0 | 4.9 | 1152.3 |
| 49 | 4732.6 | 31900.0 | 0.0 | 4.8 | 1358.0 | 5041.2 | 17600.0 | 0.0 | 3.1 | 925.9 |
| 48 | 5103.0 | 28600.0 | 0.0 | 5.8 | 1460.9 | 5205.8 | 29700.0 | 0.0 | 6.0 | 1275.7 |
| 47 | 4917.8 | 16500.0 | 0.0 | 4.6 | 1111.1 | 5329.3 | 46200.0 | 0.0 | 13.9 | 2140.0 |
| 46 | 4670.9 | 12100.0 | 0.0 | 2.6 | 720.2 | 5123.5 | 47300.0 | 0.0 | 6.9 | 1522.7 |
| 45 | 4609.1 | 24200.0 | 0.0 | 5.1 | 1440.4 | 5473.3 | 44000.0 | 0.0 | 10.9 | 1995.9 |
| 44 | 4526.8 | 16500.0 | 0.0 | 4.7 | 1172.9 | 5082.4 | 37400.0 | 0.0 | 7.1 | 1666.7 |
| 43 | 4465.1 | 17600.0 | 0.0 | 3.3 | 967.1 | 5041.2 | 30800.0 | 0.0 | 5.6 | 1399.2 |
| 42 | 4423.9 | 18700.0 | 0.0 | 4.4 | 1275.7 | 5020.7 | 37400.0 | 0.0 | 7.1 | 1934.2 |
| 41 | 4341.6 | 25300.0 | 0.0 | 5.7 | 1440.4 | 5103.0 | 35200.0 | 0.0 | 18.5 | 2242.8 |
| 40 | 4259.3 | 15400.0 | 0.0 | 3.9 | 1090.6 | 4547.4 | 29700.0 | 0.0 | 8.4 | 1687.3 |
| 39 | 4259.3 | 16500.0 | 0.0 | 5.8 | 1522.7 | 4526.8 | 47300.0 | 0.0 | 13.7 | 2469.2 |
| 38 | 4115.3 | 36300.0 | 0.0 | 7.1 | 1749.0 | 4053.6 | 29700.0 | 0.0 | 11.9 | 1666.7 |
| 37 | 4238.7 | 35200.0 | 0.0 | 6.7 | 1543.2 | 3991.8 | 33000.0 | 0.0 | 7.9 | 1707.8 |
| 36 | 4423.9 | 44000.0 | 0.0 | 8.1 | 2140.0 | 4053.6 | 57200.0 | 0.0 | 12.9 | 2345.7 |
| 35 | 4547.4 | 47300.0 | 0.0 | 8.3 | 1975.3 | 4012.4 | 45100.0 | 0.0 | 9.7 | 1913.6 |
| 34 | 4794.3 | 35200.0 | 0.0 | 7.9 | 2037.1 | 4135.9 | 40700.0 | 0.0 | 8.4 | 1749.0 |
| 33 | 4588.5 | 66000.0 | 10.3 | 12.9 | 2839.5 | 4362.2 | 47300.0 | 0.0 | 11.6 | 2325.1 |
| 32 | 4732.6 | 64900.0 | 20.6 | 12.5 | 2880.7 | 4423.9 | 69300.0 | 20.6 | 13.2 | 2716.1 |
| 31 | 4691.4 | 44000.0 | 0.0 | 8.0 | 2181.1 | 4670.9 | 78100.0 | 20.6 | 17.8 | 2880.7 |
| 30 | 4712.0 | 81400.0 | 20.6 | 16.2 | 3436.3 | 4568.0 | 82500.0 | 20.6 | 19.2 | 2963.0 |
| 29 | 4794.3 | 74800.0 | 20.6 | 13.4 | 3024.7 | 4691.4 | 107800.0 | 20.6 | 26.1 | 3498.0 |
| 28 | 4938.3 | 94600.0 | 20.6 | 18.7 | 3518.6 | 5144.1 | 128700.0 | 20.6 | 22.0 | 3868.4 |
| 27 | 5144.1 | 117700.0 | 20.6 | 21.7 | 3950.7 | 5432.2 | 114400.0 | 20.6 | 22.5 | 4012.4 |
| 26 | 5329.3 | 174900.0 | 20.6 | 42.4 | 4465.1 | 5185.3 | 139700.0 | 20.6 | 27.1 | 4362.2 |
| 25 | 5288.1 | 168300.0 | 20.6 | 29.6 | 4547.4 | 5247.0 | 174900.0 | 35.6 | 42.4 | 4609.1 |
| 24 | 6008.3 | 178200.0 | 20.6 | 31.6 | 5308.7 | 5103.0 | 169400.0 | 20.6 | 31.3 | 4177.0 |
| 23 | 5967.2 | 195800.0 | 35.6 | 30.4 | 5432.2 | 5699.7 | 172700.0 | 20.6 | 32.5 | 4814.9 |
| 22 | 6461.0 | 226600.0 | 35.6 | 34.8 | 5905.4 | 6193.5 | 229900.0 | 35.6 | 33.3 | 5720.3 |
| 21 | 6378.7 | 203500.0 | 35.6 | 34.4 | 6090.6 | 6769.7 | 190300.0 | 20.6 | 29.0 | 5823.1 |
| 20 | 6193.5 | 216700.0 | 35.6 | 32.5 | 5802.6 | 6378.7 | 177100.0 | 20.6 | 29.8 | 5617.4 |
| 19 | 6831.4 | 292600.0 | 35.6 | 40.5 | 6646.2 | 6214.1 | 204600.0 | 20.6 | 27.2 | 5411.6 |
| 18 | 7448.7 | 277200.0 | 35.6 | 50.5 | 7016.6 | 7592.7 | 217800.0 | 20.6 | 30.7 | 6769.7 |
| 17 | 6028.9 | 207900.0 | 35.6 | 37.3 | 5864.3 | 7777.9 | 283800.0 | 35.6 | 34.8 | 7078.3 |
| 16 | 5864.3 | 207900.0 | 35.6 | 38.3 | 5535.1 | 8518.6 | 297000.0 | 35.6 | 33.3 | 7736.7 |
| 15 | 5823.1 | 224400.0 | 35.6 | 44.0 | 5576.2 | 9424.0 | 332200.0 | 35.6 | 38.1 | 8765.6 |
| 14 | 5884.9 | 233200.0 | 35.6 | 44.0 | 5740.8 | 9259.4 | 336600.0 | 35.6 | 50.0 | 8889.0 |
| 13 | 5884.9 | 232100.0 | 35.6 | 43.6 | 5720.3 | 8703.8 | 345400.0 | 35.6 | 42.3 | 8251.2 |
| 12 | 5946.6 | 246400.0 | 35.6 | 44.2 | 5843.7 | 8724.4 | 289300.0 | 20.6 | 36.4 | 7901.4 |
| 11 | 6111.2 | 260700.0 | 35.6 | 43.0 | 5946.6 | 8415.8 | 257400.0 | 20.6 | 29.9 | 7736.7 |
| 10 | 6522.7 | 225500.0 | 35.6 | 37.5 | 6378.7 | 8683.3 | 328900.0 | 35.6 | 37.6 | 8292.3 |
| 9 | 6502.2 | 234300.0 | 35.6 | 36.1 | 6234.7 | 9362.3 | 502700.0 | 54.4 | 74.6 | 9156.5 |
| 8 | 6893.1 | 239800.0 | 35.6 | 36.2 | 6646.2 | 8395.2 | 281600.0 | 35.6 | 50.4 | 7757.3 |
| 7 | 6934.3 | 233200.0 | 35.6 | 36.8 | 6769.7 | 6605.0 | 265100.0 | 35.6 | 71.4 | 6543.3 |
| 6 | 6522.7 | 122100.0 | 20.6 | 18.4 | 5061.8 | 5432.2 | 112200.0 | 20.6 | 23.8 | 4300.5 |
| 5 | 6502.2 | 187000.0 | 20.6 | 29.6 | 5864.3 | 4979.5 | 194700.0 | 35.6 | 45.4 | 4958.9 |
| 4 | 6317.0 | 112200.0 | 20.6 | 16.3 | 4465.1 | 4465.1 | 106700.0 | 20.6 | 24.9 | 3786.1 |
| 3 | 6193.5 | 108900.0 | 20.6 | 17.6 | 4279.9 | 4238.7 | 128700.0 | 35.6 | 32.4 | 3909.5 |
| 2 | 5926.0 | 101200.0 | 20.6 | 15.6 | 4177.0 | 4033.0 | 223300.0 | 54.4 | 60.1 | 4033.0 |
| 1 | 5823.1 | 71500.0 | 0.0 | 11.5 | 3333.4 | 3600.9 | 190300.0 | 35.6 | 42.5 | 3148.2 |
