# Supplementary material for: Quantifying the spatiotemporal dynamics of the first two epidemic waves of SARS-CoV-2 infections in the United States
Source: PLoS Comput Biol. 2026 Mar 4;22(3):e1013983. doi: 10.1371/journal.pcbi.1013983 (PMC12959703; doi:10.1371/journal.pcbi.1013983)
Supplement: Attachment — Submitted filename: response_Letter-Waves.docx [file pcbi.1013983.s012.docx]

We thank the editor and reviewers for the opportunity to address comments and revise our manuscript for consideration at PLOS Computational Biology. All line numbers below refer to the lines of the new clean version of the manuscript. All blue text has been inserted into the manuscript.

Due to the required extensive changes to the manuscript and increased responsibilities taken by author Nicole A. Swartwood, this author has been updated to be the senior author on the paper. All current authors were included on the original manuscript, we’ve only moved the order to accommodate N. Swartwood’s senior authorship.

Journal Requirements

1) We ask that a manuscript source file is provided at Revision. Please upload your manuscript file as a .doc, .docx, .rtf or .tex. If you are providing a .tex file, please upload it under the item type u2018LaTeX Source Fileu2019 and leave your .pdf version as the item type u2018Manuscriptu2019.

Thank you, we have uploaded the source file as part of the revision.

2) Please upload all main figures as separate Figure files in .tif or .eps format. For more information about how to convert and format your figure files please see our guidelines:
<https://journals.plos.org/ploscompbiol/s/figures>. Please also ensure that figure 3 is labeled.

We have corrected the labeling of all figures.

3) We have noticed that you have uploaded Supporting Information files, but you have not included a complete list of legends. Please add a full list of legends for your Supporting Information files (Supplementary Movies) after the references list.

We have added a list of legends to the supporting material file.

4) Some material included in your submission may be copyrighted. According to PLOSu2019s copyright policy, authors who use figures or other material (e.g., graphics, clipart, maps) from another author or copyright holder must demonstrate or obtain permission to publish this material under the Creative Commons Attribution 4.0 International (CC BY 4.0) License used by PLOS journals. Please closely review the details of PLOSu2019s copyright requirements here: PLOS Licenses and Copyright. If you need to request permissions from a copyright holder, you may use PLOS's Copyright Content Permission form.
Please respond directly to this email and provide any known details concerning your material's license terms and permissions required for reuse, even if you have not yet obtained copyright permissions or are unsure of your material's copyright compatibility. Once you have responded and addressed all other outstanding technical requirements, you may resubmit your manuscript within Editorial Manager.
Potential Copyright Issues:
i) Figures 1, 2, S3, S4, and S5. Please (a) provide a direct link to the base layer of the map (i.e., the country or region border shape) and ensure this is also included in the figure legend; and (b) provide a link to the terms of use / license information for the base layer image or shapefile. We cannot publish proprietary or copyrighted maps (e.g. Google Maps, Mapquest) and the terms of use for your map base layer must be compatible with our CC BY 4.0 license.
Note: if you created the map in a software program like R or ArcGIS, please locate and indicate the source of the basemap shapefile onto which data has been plotted.
If your map was obtained from a copyrighted source please amend the figure so that the base map used is from an openly available source. Alternatively, please provide explicit written permission from the copyright holder granting you the right to publish the material under our CC BY 4.0 license.
If you are unsure whether you can use a map or not, please do reach out and we will be able to help you. The following websites are good examples of where you can source open access or public domain maps:
* U.S. Geological Survey (USGS) - All maps are in the public domain. (<http://www.usgs.gov>)
* PlaniGlobe - All maps are published under a Creative Commons license so please cite u201cPlaniGlobe, <http://www.planiglobe.com>, CC BY 2.0u201d in the image credit after the caption. (<http://www.planiglobe.com/?lang=enl>)
* Natural Earth - All maps are public domain. (<http://www.naturalearthdata.com/about/terms-of-use/>).

We have fixed all the figure legends to reflect the data sources for public domain maps and also added the following text to our data availability section (lines 145-147):

“All the maps were generated using United States, state, and county borderlines maps in public domain from the Census Bureau which were downloaded through the R package Tigris (35).”

5) We note that your Data Availability Statement is currently as follows: "All relevant data are within the manuscript and its Supporting Information files." Please confirm at this time whether or not your submission contains all raw data required to replicate the results of your study. Authors must share the “minimal data set” for their submission. PLOS defines the minimal data set to consist of the data required to replicate all study findings reported in the article, as well as related metadata and methods (<https://journals.plos.org/plosone/s/data-availability#loc-minimal-data-set-definition>).
For example, authors should submit the following data:
1) The values behind the means, standard deviations and other measures reported;
2) The values used to build graphs;
3) The points extracted from images for analysis..
Authors do not need to submit their entire data set if only a portion of the data was used in the reported study.
If your submission does not contain these data, please either upload them as Supporting Information files or deposit them to a stable, public repository and provide us with the relevant URLs, DOIs, or accession numbers. For a list of recommended repositories, please see <https://journals.plos.org/plosone/s/recommended-repositories>.
If there are ethical or legal restrictions on sharing a de-identified data set, please explain them in detail (e.g., data contain potentially sensitive information, data are owned by a third-party organization, etc.) and who has imposed them (e.g., an ethics committee). Please also provide contact information for a data access committee, ethics committee, or other institutional body to which data requests may be sent. If data are owned by a third party, please indicate how others may request data access.

We have updated our data availability statement (lines 273-282):

“Daily county estimates of per capita infections used in the analysis are available at Harvard Dataverse (58). We have made our repository, <https://github.com/covidestim/waves>, with the relevant scripts and necessary origination data, such as hexagonally distributed infections per capita, publicly available. These materials allow the reproduction of all the figures and analytical products in this analysis. We will also make a static repository of all codes and intermediary data products available on Zenodo once accepted for publication and will include the relevant DOI information in the text at that time.”

6) Please amend your detailed Financial Disclosure statement. This is published with the article. It must therefore be completed in full sentences and contain the exact wording you wish to be published.
1) State the initials, alongside each funding source, of each author to receive each grant. For example: "This work was supported by the National Institutes of Health (####### to AM; ###### to CJ) and the National Science Foundation (###### to AM)."
2) State what role the funders took in the study. If the funders had no role in your study, please state: "The funders had no role in study design, data collection and analysis, decision to publish, or preparation of the manuscript."

We corrected the Financial Disclosure statement by adding which authors were financed by which contract (lines 268-275):

**“Funding**

This project is supported by Cooperative Agreement NU38OT000297 from the Centers for Disease Control and Prevention (CDC), SHEPheRD Contract 200-2016-91779 from the CDC, and the CDC Broad Agency Announcement Contract 75D30122C14697 (RL, YL, MHC, NDG, JLW, and TC) and the Council of State and Territorial Epidemiologists (CSTE) (NAS, FK, JAS, TC and NAM). This work does not necessarily represent the views of the CDC or CSTE. The funders had no role in study design, data collection and analysis, decision to publish, or preparation of the manuscript. The authors received no specific funding for this work”

7) Please ensure that the funders and grant numbers match between the Financial Disclosure field and the Funding Information tab in your submission form. Note that the funders must be provided in the same order in both places as well." Currently, "Council of State and Territorial Epidemiologists (CSTE)" is missing from the Funding Information tab.

Thank you, we have checked and corrected this text.

Reviewers' comments:

**Reviewer#1**

The authors present a very interesting work on the speed of SARS-CoV-2 spread across the United States. The work is of interest to the wider public as (i) it can be used to inform public policy both in terms of surveillance and response and (ii) it can easily be applied to other viruses as such methodologies are highly scarce. However, a few things need more clarity and attention, as the manuscript in very succinct at times.

We thank the reviewer for the constructive and helpful comments. We believe that our changes summarized in the responses below improve the clarity of the manuscript.

1- It would be good to include some information in the introduction about the different waves in the US: epidemiological scenario, variants, interventions, etc. This is really important to understand the results and it is only really brought up in the discussion.

We added a new paragraph to the introduction (lines 57-36):

“The two major epidemic waves we analyzed overlap with the introduction of the ‘Alpha’ (B.1.1.7) variant of concern (VOC) in mid-September 2020 and the ‘Delta’ (B.1.617.2) VOC in early July 2021 (14–18). We note however, that the first wave we study begins to rise in advance of the widespread emergence of the Alpha VOC, hence we refer to these waves as Wave 1 and Wave 2, as they do not perfectly align with a singular genetic variant in circulation. (17–20). These waves spread within distinct epidemiological scenarios with respect to intervention policies and population susceptibility due to previous infection and vaccination.”

2- It is not clear to me how the waves were selected. I do understand that a threshold for the number of infections was used for that. However, wave 1 is only considered to have started in September 2020, when the wildtype was already circulating in the united states for at least 7 months. As a reader, it would have been more interesting to see a characterisation of the epidemic in the country since its introduction and first original wave back in March. This would reveal an interesting perspective on the role of different areas of the country in spreading the virus in a completely naive population, which is not true for the two waves analysed here. It is also confusing that the large wave in September is called wave 1 when from the plots there were two prior waves, much smaller, but still very epidemiologically important at the time. For instance, a close look at the very first wave March to June could have revealed the potential impact of interventions in decreasing the spread through a potential reduction in the speed.

We thank the reviewer for this comment. We took a purely quantitative approach (i.e. based on estimated infection rates) for defining waves as we wanted to develop an approach that was agnostic of variant type. While we agree that the wild-type peaks are worthy of analysis, they did not meet the criteria adopted for this analysis, as they were too small. We hope that future work can be done to expand on this analytical approach that may make it more appropriate for analyzing the wild type peaks.

We have added a new section to the methods section that describes the definition of a wave (lines 115-128):

**“Definition of a wave**

To formally determine hexagons that are within a wave, we defined a threshold of per-capita SARS-CoV-2 infection above which we coded hexagons as being part of an infection wave. For our main analysis, we used a threshold of 165 daily infections per capita to characterize a hexagon as being within a wave. This value represents the 75^th^ percentile of the infections-per-capita value distribution during the study period (**Figure S1**). We conducted sensitivity analyses using 85 and 300 infections per capita as alternative threshold values (**Supplementary Material Figure S2)**. To allow for multiple infection centers, we did not limit our definition to a single contiguous set of hexagons.

Using this definition, we categorized Wave 1 as the sets of contiguous hexagons above the threshold within the period ranging from September 2020 to February 2021, and Wave 2 as the sets of contiguous hexagons above the 165 infections per capita threshold during the period from July 2021 to November 2021. We report the epicenter of each wave by visual inspection.”

3- A suggestion could also be to define waves based on variant surveillance. For instance, it is assumed that both waves started from one focus of spread. However, while the second wave probably overlaps with the introduction of delta, wave 1 had already seen 7 months of wildtype spread. A model using genomic data and case counts could help pinpoint the start of a new way. (not mandatory)

We thank for the comment. We are also interested in using the genomic information from surveillance data to help define and characterize waves of infections. However, genomic surveillance may not be a sufficient criterion for spatial wave definition, as the sequencing effort varied over time and by location. For another analysis, unrelated to this paper, we produced the following map showing the disparities in surveillance between the state level and the county level in the US. Though almost all states had state-level representative efforts on genomic surveillance for SARS-CoV-2, this is not the case at the county level. The variability of sequencing count and bias towards more populated regions within states is strikingly . See the following figure:

Figure 1 - Counts of SARS-CoV-2 genomic sequences with county-level information per county, from March 2020 to October 2024. Out of a total of roughly ~5 million sequences available for this time period, 100% had state-level information, while only ~1 million had county-level information. Grey-filled counties had no sequences ever recorded. Metadata was gathered from GISAID.

4- While from the maps it is very clear that wave 1 did start in the midwest and then radially spread across the country, I believe that wave 2 is better described as a wave caused by several origins, which could well describe a wave caused by the introduction of a new variant in multiple areas or with multiple variants circulating in different areas of the country. As noted in figures 2F-H there seems to have been multiple origins for wave two: the Ozarks, Wyoming, Nevada and Oregon.

We agree with the reviewer that wave 2 had multiple origins within the US. As mentioned in response to comment #2, we have added further language in the methods to clarify how we defined a wave, specifically addressing the potential for multiple wave origins (lines 122-123):

"To allow for multiple infection centers, we did not limit our definition to a single contiguous set of hexagons.”

We have also revised the results (lines 161-165) to state that wave 2 was characterized by multiple starting points:

“Wave 2 originated in the Ozarks (southern Missouri and northern Arkansas) (**Figure 2F**) in early July 2021 and then expanded further south (**Figure 2G-I**). Secondary centers of infection appeared later in July in the Pacific Northwest (**Figure 2G**), and the infection wave subsequently spread throughout the western United States (**Figure 2G-I**).”

We also edited the discussion to reflect the difference in origins between the two waves. (lines 211-225):

“Our findings suggest that the first and second waves differed in both their origin and expansion patterns. Wave 1 originated in parts of northeastern Montana, eastern North Dakota, and central South Dakota in September 2020 (**Figures 2B-E** and **Movie 2**). The estimated point of origin coincides with a motorcycle rally that brought over 460,000 attendees to Sturgis, South Dakota in August 2020 and was later epidemiologically implicated in elevated local COVID-19 rates and interstate spread of infection (31,32).

In contrast, Wave 2 involved multiple epicenters, beginning first in the Ozarks (**Figures 2F** and **Movie 2**), during a time when there was local re-opening of indoor music venues and relaxation of local masking policies (33–35). Shortly after, secondary high-incidence centers appeared in the Pacific Northwest. The decentralized and near-simultaneous emergence of multiple hotspots during Wave 2 aligns with earlier work demonstrating that both domestic and international travel networks have facilitated the rapid dispersal of viruses (7,36,37). Wave 2 coincided with the emergence and national spread of the Delta variant (B.1.617.2), which became dominant in the United States during this period (18).”

5- Speed or velocity of spread are normally represented in km/day as they describe the movement in linear direction rather than area, you would probably be more appropriate to either represent speed as km/day, or call the measure being report as a diffusion coefficient or rate, km2/day.

We thank the reviewer for noticing this inconsistency and we agree with the comment. We have applied new quantitative approaches to calculate both the areal expansion of each wave, measured in km^2^/day, and wavefront speed, measured in km/day. We have added language to the methods section lines (130-143) describing these new calculations:

**“Calculation of wavefront speed and areal wave expansion**

We first identified the leading edge, or wavefront, of each wave. This wavefront was defined as the two-dimensional boundary between hexagons included and excluded from a wave on each day of the analysis. After delineating the wavefront, we estimated two quantities: the daily areal wave expansion and the daily wavefront speed. To calculate the areal wave expansion rate, we tracked the daily change in the number of hexagons (or area) included in a wave. This quantity is the difference between the area of the wave at day *d+1* and day *d*, giving an areal wave expansion rate measured in km^2^/day.

To estimate the wavefront speed, we identified the mid-point of each line-segment comprising the wavefront at each day, *d.* We calculated the distance between the midpoint of each line-segment and the nearest point on subsequent wavefront at day, *d+1*, which gives us a measure of distance travelled per day (km/day), or speed for each segment of the wavefront. This allows us to capture the heterogeneity in speed along the length of the wavefront.”

6- 580,000 km2/day would be around 760 km/day. This would be equivalent to spread form New York to Columbus in one single day. Although that is probably correct due to the high aerial connectivity in the USA, I think it would be probably more interesting to understand what the median and mean speed of spread/diffusion coefficients are as spread can be highly skewed and long-tailed, with long distance movements having some impact but normally less frequency than short distance movements. What is the distribution of the estimates for the wavefront velocity increase? Finally, how did the authors handle outliers, i.e., days in which the spread was extremely high or extremely low?

We thank the reviewer for this comment and suggestion. As mentioned in the response to reviewer’s comment #5, we now calculate speed of the wavefront. We have presented additional results describing the wavefront speed with an emphasis on median values. We also provide the range of each wave to show the potential for wavefront speeds that were extremely high or extremely low (lines 178-196):

**“Wave speed**

The overall median wavefront speed of Wave 1 was of 149 km/day across the 63 days of analysis (daily median wavefront speed range: 0 to 5,048 km/day). Wave 2 had a higher overal median wavefront speed of 748 km/day (range from 0 to 4,850 km/day). **Table S5** provides daily median wavefront speeds for each wave.

There was heterogeneity in the estimated daily wavefront speed across the edge of each wave. **Figure 4** shows the speed each wave at four dates leading up to each wave’s infection peak. Because each infection peak occurred after the wave had spread across the contiguous United States, the final date shown is the last day of wavefront progression: November 1, 2020 for Wave 1 and August 19, 2021 for Wave 2.

Because wavefronts varied in length, the percentage of the wavefront at maximal speed changed over time; to quantify this change we calculated the length of the wavefront exceeding the median maximum speed over the study period. At its longest, the wavefront of Wave 1 had 5,934 kilometers moving at speed greater than or equal to the overall median maximal speed at 27 days prior to its infections per capita peak, while Wave 2 had 9,341 kilometers at 21 days prior to its peak. Daily wavefront lengths moving at speeds exceeding the median maximum are in **Table S5**.”

We have also added an additional table to the supplement which presents the daily median speed for each wave and Figure 4 allows the reader to see the heterogeneity in the speed over time.

7- Although the speed of spread is the main finding in the manuscript, there is little description on how the calculation was actually performed. For instance, how was distance measured here? What kind of distance is being used, geodesic? Which point in the grid was used, centroid?

We thank the reviewer for the comment. As mentioned in the response to comment #5, we now include new results which estimate two distinct quantitative measures of wave movement: wavefront speed and areal wave expansion rate. To calculate the wavefront, we use the mid-point [centroid] of each line segment [hex side] that forms the boundary between non-wave and wave hexes. The full description of this calculation can be found in the Methods and Materials section of the manuscript, lines 132-136:

“To estimate the wavefront speed, we identified the mid-point of each line-segment comprising the wavefront at each day, *d.* We calculated the distance between the midpoint of each line-segment and the nearest point on subsequent wavefront at day, *d+1*, which gives us a measure of distance travelled per day (km/day), or speed for each segment of the wavefront. This allows us to capture the heterogeneity in speed along the length of the wavefront.”

8- Were there any regional differences in the speed of spread? Maps coloured by the speed of spread/diffusion rate would give us a greta information on local variation in virus spread.

We thank the reviewer for the comment and we have added a new figure, Figure 4, and new movies, Movies “FirstWavefrontSpeed.mov and SecondWavefrontSpeed.mov, to address the suggestion. Each of these visualizations show the daily wavefront colored according to its speed. These figures allow us to capture the the heterogeneity in wavefront speed over the analysis period.

For example, in Figure 4 panel labelled “2020-10-29" we estimate wavefront speed varying from 100-200 km/day on the western wave front while the eastern wavefront has more heterogeneity, with speed increasing as we move south along the wavefront. We also see varying wavefront speeds in the movies.

9- Finally, while the text refers to several panels within figure 3, there is only one graph being presented in the actual figure. Probably the other figures would be needed to understand the full context of the manuscript.

We apologize to the reviewer as we incorrectly referred to panels in Figure 2 as panels in Figure 3. We have corrected this error and have reviewed and updated all figure referencing throughout the manuscript.

**Reviewer#2**

Reviewer #2: In their article, López et al. present an innovative approach to mapping the spatial spread of SARS-CoV-2 infections across the United States, using the BYM2 model to generate daily infection per capita surfaces, which were later used to calculate the speed of wave expansion. Overall, I found the article well-written and easy to follow. The methodology is clearly described, and the visualizations—both the plots and movies—are nicely done and effective in illustrating their results.

We thank the reviewer for these kind comments and hope that our responses and edits below address the reviewer’s comments below.

However, I have a few minor comments:

1. The authors used an area-weighted approach to distribute population estimates from CBGs to hexagons, assuming a constant population throughout the analysis period. However, could they have instead used a population raster, with high-resolution gridded population data, to assign a more precise and spatially realistic population estimate per hexagon? Same thing with the estimates of infections. Why did you choose an area-weighted approach?

We thank the reviewer for this comment which led to major changes in our approach. We have now used the Meta 30m population grid data, which has population estimates at 30m^2^ resolution. A comparison of population distribution from our previous approach using census block group data and this new approach using the Meta population data are shown below:

Figure 2 - Comparison of population allocated using Census Block Groups and Meta 30m^2^ raster .

For infection estimates, we are restricted to using county-level geography, as case notifications are only available at this resolution.

We thank the reviewer for raising this important potential source bias in our analysis. We chose area weighted because we felt the computational efficiency and transparency of the approach offset any potential bias from assuming uniform distribution of population. Following the reviewers’ suggestion we revised our approach to use a more granular population source, specifically the Meta 30m^2^ population estimates. Now that we are using an even more granular population source, we believe the probability of this bias is even further reduced.

2. In lines 178-186 the authors note that while Wave2 showed a higher, but overall similar, maximal speed of expansion than Wave1, they argue that human mobility played a more significant role than viral transmissibility, immunity, and policy changes. However, given that (i) multiple studies have demonstrated that the Delta variant is more transmissible than both the wild-type and Alpha variants, (ii) international and domestic travel restrictions were more relaxed in Wave 2 compared to Wave 1, (iii) NPIs were also more relaxed during the Wave 2, shouldn’t the Wave2 have spread more rapidly then as written in lines 33-34? Maybe the lack of substantial difference in the speed of expansion could be attributed to the (likely) increased vaccine coverage and naturally acquired immunity in Wave2? Could the authors clarify the reasoning behind their conclusion that human mobility had a stronger influence than these other variables?

We thank and agree with the reviewer comments. We moderate the language we use when talking about the impact of mobility, and we added as explanatory factors those mentioned by the reviewer. Now the part in the Discussion section [lines 227-239] reads as follows:

“While we found that Wave 2 had both a higher maximal speed of wavefront movement and achieved its largest areal expansion more quickly than Wave 1, the similarity in estimates of speed of wavefront movement and the growth rate in wave size for these first two waves is striking. This similarity is remarkable considering the distinct virological, immunological, and policy contexts in which each wave occurred. Wave 1 was due to wildtype SARS-CoV-2 as well as the ‘Alpha’ variant (38,39) while Wave 2 was due to the ‘Delta’ variant (18,40) . Regional and secular differences in non-pharmaceutical intervention policies, and differences in the levels and types of infection- and vaccine-induced population immunity at the time of these two waves (41), makes the similarity between the movement patterns of these waves particularly surprising. adds explanatory factors to the stability observed in diffusion and speed of spread of each wave. Such stability may suggest that underlying patterns of human mobility may have had a stronger impact on the patterns of local epidemic expansion than only virological, non-pharmaceutical interventions, and host-immunity factors (7,32).”

3. The GitHub repository does not appear to be accessible, please make it available before publication.

We made the repository publicly available. The repo is available at: <https://github.com/covidestim/waves>.

**Reviewer #3**

\# Overall Assessment

This manuscript presents a quantitative analysis of the spatiotemporal dynamics of the first two major COVID-19 infection waves in the United States. The study employs a BYM2 spatial model to estimate an infection surface and uses it to evaluate the speed and spatial extent of viral spread. The manuscript is well-structured, and the methods are sound. However, the analysis is broad and lacks deeper insights. Some areas require further clarification and improvement, as outlined below.

We thank the reviewer for these comments and hope our manuscript edits and responses below increase the clarity of our report.

\# Key Findings

The research provides effective visualizations of the spread of SARS-CoV-2. According to the Author Summary, this is the main goal of the study. The authors also produce global estimates for disease wave spread speed during two major waves in the U.S.

We thank the reviewer for this comment.

\# Strengths

- The visualizations are clear and well-executed.

We thank the reviewer for this kind comment.

\# Weaknesses

- The definitions of wave and speed are vague.
- The concept of speed, as derived, has limited applicability.

We have revised our definition of wave speed, described in detail below. We believe the associated updates improves the applicability of our analysis.

\# Specific Comments

- I could not access the GitHub repository: [[https://github.com/covidestim/waves](https://github.com/covidestim/waves)](https://github.com/covidestim/waves%5D(https://github.com/covidestim/waves)).

We made the repository publicly available and pinned the branch with the code to reproduce the analysis. Please see <https://github.com/covidestim/waves>.

Line 33-34: "driven by multiple, non-contagious sources of infection" – A source is needed to support this statement.

We thank the reviewer for this comment. This phrase was written as a finding of the paper. To clarify, we have revised the statement (lines 31-32):

“Notably, we found that the second wave spread more rapidly than the first, possibly driven by multiple introduction events.”

Line 54: "Efforts to quantify the speed and spatial extent of waves have been limited" – While this is true, there have been attempts to quantify disease spread. Some of these studies focus on point pattern analysis of disease spread, which is worth mentioning. Some are mentioned at the end of this review.

\# Additional Methodological References

In point pattern models using partial differential equations (PDEs), the definition of velocity is straightforward. Some relevant works on estimating disease spread through point patterns include:
- Velocities for spatio-temporal point patterns ([[https://doi.org/10.1016/j.spasta.2018.12.007](https://doi.org/10.1016/j.spasta.2018.12.007)](https://doi.org/10.1016/j.spasta.2018.12.007%5D(https://doi.org/10.1016/j.spasta.2018.12.007)))
- Estimating velocities of infectious disease spread through spatio-temporal log-Gaussian Cox point processes ([[https://arxiv.org/abs/2409.05036](https://arxiv.org/abs/2409.05036)](https://arxiv.org/abs/2409.05036%5D(https://arxiv.org/abs/2409.05036)))

A related approach to the authors’ method is trend-surface analysis (TSA), where a surface is fitted to reported cases and speeds are estimated from the surface. Relevant studies include:
- Estimating front-wave velocity of infectious diseases: a simple, efficient method applied to bluetongue ([[https://doi.org/10.1186/1297-9716-42-60](https://doi.org/10.1186/1297-9716-42-60)](https://doi.org/10.1186/1297-9716-42-60%5D(https://doi.org/10.1186/1297-9716-42-60)))
- Spatial diffusion of raccoon rabies in Pennsylvania, USA ([[https://doi.org/10.1016/s0167-5877(99)00005-7](https://doi.org/10.1016/s0167-5877(99)00005-7](https://doi.org/10.1016/s0167-5877(99)00005-7%5D(https://doi.org/10.1016/s0167-5877(99)00005-7)))

Other relevant works can be found within the references.

We expanded the phrase to better contextualize our work within the disease mapping literature, and we also cite the mentioned work. Now the phrase in lines 251-60 reads as follows:

“Factors that affect the diffusion and speed of epidemic waves is an area of substantial interest for other viral diseases (43) like influenza (44-46), Ebola (e.g. (47), HIV (48), measles (49), and parasitic diseases such as malaria (50). While our work on SARS-CoV-2 does not provide explanations for why each of these first two epidemic waves spread in the manner that they did once they were established, the development of methods to quantify the wavefront speed and rate of areal expansion of epidemic waves, such as those we employed here, are a necessary first step. Related work has evaluated the quantification of wave dynamics for point pattern disease data (51–53) and from trend surface analysis (54,55).”

Line 80: "each enclosing 64.75 square kilometers" – The reason for this choice should be explained. Is it based on a minimum area threshold for U.S. census block groups (CBGs)? Are the estimates sensitive to this choice?

We thank the reviewer for raising this question. Our choice was based on a balance between the total number of hexes to tile the contiguous US and a deliberate choice in having a smaller unit than the typical size of a county polygon. We added the following phrase to the Materials and Methods section at lines 87-89:

“The grid is comprised of 7,665 unique hexagons, each enclosing 64.75 square kilometers (25 square miles). This hexagon size is smaller than the mean size of counties but still large enough to permit a national analysis.”

Line 81: "We employed an area-weighted approach" – Area weighting might introduce bias since areas with little or no population may receive a disproportionate share of the estimate. Consider referencing ESRI's methodology for population estimation based on land-use data, which classifies land from densely populated to rural and distributes census estimates accordingly. This would likely produce more reliable population estimates.

We thank the reviewer for raising this important potential source bias in our analysis. We chose area weighted because we felt the computational efficiency and transparency of the approach offset any potential bias from assuming uniform distribution of population. Following the reviewers’ suggestions we revised our approach to use a more granular population source, specifically the Meta 30m2 population estimates. Now that we are using an even more granular population source, we believe the probability of this bias is even further reduced.

Line 97-98: "for more details on the priors used, see the Supplementary Material" – The supplementary material only states that PC-priors were used. If the hyperparameter selection followed INLA’s default choice, this should be explicitly mentioned.

We updated the supplementary material to state correctly which hyperparameters we employed in the analysis.

Now the line 46 in the Supplementary Material reads as follows:

“We used INLA’s default choice for the priors.”

Line 107: The definition of wave is vague. The study defines waves spatially, yet Wave 1 and Wave 2 are described in temporal terms. The definition should encompass both spatial and temporal characteristics.

We now define a wave via both temporal and spatial characteristics.

Materials and Methods now have a dedicated section called ‘Definition of a wave’ from lines 116 - 123 that reads as follows:

**“Definition of a wave**

To formally determine hexagons that are within a wave, we defined a threshold of per-capita SARS-CoV-2 infection above which we coded hexagons as being part of an infection wave. For our main analysis, we used a threshold of 165 daily infections per capita to characterize a hexagon as being within a wave. This value represents the 75^th^ percentile of the infections-per-capita value distribution during the study period (**Figure S1**). We conducted sensitivity analyses using 85 and 300 infections per capita as alternative threshold values (**Supplementary Material Figure S2)**. To allow for multiple infection centers, we did not limit our definition to a single contiguous set of hexagons.”

Line 118: The definition of speed is simplistic and may not be informative. Specifically:
- The definition of speed is global, which presents issues. As the visualizations and author comments indicate, regional heterogeneity is of great interest. Global speed estimates do not provide insight into local events.
- Events such as the motorcycle rally (Line 165) and the relaxation of mask policies (Line 169) should be examined in the context of local disease spread estimates.
- Notably, the manuscript does not use speed estimates to derive meaningful conclusions. Speeds are merely computed and compared to the national curve peak and to one another.

We have revised our definition and calculation of speed in the analysis. We now calculate the speed of the wave front expansion. We use the mid-point of each line segment that comprises the boundary between non-wave and wave hexes. The full description of this calculation can be found in the Methods and Materials section of the manuscript, lines 130-143:

**“Calculation of wavefront speed and areal wave expansion**

We first identified the leading edge, or wavefront, of each wave. This wavefront was defined as the two-dimensional boundary between hexagons included and excluded from a wave on each day of the analysis. After delineating the wavefront, we estimated two quantities: the daily areal wave expansion and the daily wavefront speed. To calculate the areal wave expansion rate, we tracked the daily change in the number of hexagons (or area) included in a wave. This quantity is the difference between the area of the wave at day *d+1* and day *d*, giving an areal wave expansion rate measured in km^2^/day.

To estimate the wavefront speed, we identified the mid-point of each line-segment comprising the wavefront at each day, *d.* We calculated the distance between the midpoint of each line-segment and the nearest point on subsequent wavefront at day, *d+1*, which gives us a measure of distance travelled per day (km/day), or speed for each segment of the wavefront. This allows us to capture the heterogeneity in speed along the length of the wavefront.”

We have also added additional results regarding speed in the model into the main text lines 184-188:

**“Wave speed**

The overall median wavefront speed of Wave 1 was of 149 km/day across the 63 days of analysis (daily median wavefront speed range: 0 to 5,048 km/day). Wave 2 had a higher overal median wavefront speed of 748 km/day (range from 0 to 4,850 km/day). **Table S5** provides daily median wavefront speeds for each wave.”

We have also added an additional figure (Figure 3), additional table to the supplement which presents the daily median speed for both waves’ full analytical period.

Line 163: Regarding the estimated origins of the waves, it is important to clarify that these estimations were based on visual inspection of the graphics. No explicit coordinate-based epicenter estimation was performed.

We thank the reviewer for noting this omission. We have added language to the Methods and Materials to clarify (lines 122-123):

“We report the epicenter of each wave by visual inspection.”

Figure 1-D: Some regions have no recorded infections, but the smoothed surface produced by the BYM2 model extends disease spread into these areas, which affects the speed calculation. In a discrete spatial model, this issue can be addressed by removing these regions and updating the adjacency matrix accordingly. How would this adjustment impact the speed estimates? One possible approach to selecting regions for removal is identifying areas with dense forest coverage or sparsely populated rural zones (as mentioned in comments above).

We agree with the reviewer. When we reallocated population using the Meta 30m^2^ estimates, we removed hexes with zero population. These hexes are also not allocated any infections.

Figure S5: The figure is confusing. The description refers to "dates for the speed of expansion," but no speed information is presented. A more accurate description would be "wave boundary limits at various dates up to the national curve peak date."

We have added a new figure, Figure 4, and new movies, Movies “FirstWavefrontSpeed.mov and SecondWavefrontSpeed.mov, to address the suggestion. Each of these visualizations show the daily wavefront colored according to its speed. These figures allow us to capture the heterogeneity in wavefront speed over the analysis period.

For example, in Figure 4 panel labelled “2020-10-29" we estimate wavefront speed varying from 100-200 km/day on the western wave front while the eastern wavefront has more heterogeneity, with speed increasing as we move south along the wavefront. We also see varying wavefront speeds in the movies.
