# Supplementary material for: Quantifying the spatiotemporal dynamics of the first two epidemic waves of SARS-CoV-2 infections in the United States
Source: PLoS Comput Biol. 2026 Mar 4;22(3):e1013983. doi: 10.1371/journal.pcbi.1013983 (PMC12959703; doi:10.1371/journal.pcbi.1013983)
Supplement: Attachment — Submitted filename: 2025 - Second Rebuttal Letter - Waves.docx [file pcbi.1013983.s013.docx]

We thank the editor and reviewers for the opportunity to address comments and revise our manuscript for consideration at PLOS Computational Biology. All line numbers below refer to the lines of the new, clean version of the manuscript. All blue text has been inserted into the manuscript.

**Reviewers' comments:

Reviewer #1:**

Thank you very much for addressing the points raised in the previous round of reviews. The manuscript has clearly improved and is now much clearer. However, I still have a few concerns that I believe warrant further attention:

We thank the reviewer for their patience and attention in reading our manuscript and helping us improve it.

1-Presenting both the area and speed of expansion is a good idea. The estimated area of expansion—around 1600 km²/day or roughly 40x40 km/day—appears plausible and consistent with the figures. However, the reported speed of spread, especially for wave 2, seems too high. If speed is calculated as the straight-line distance between the wavefronts of consecutive days, then a median area of 1600 km² and a median distance of 748 km/day (wave 2) would imply a corresponding orthogonal component of only ~2 km, which seems inconsistent. Furthermore, according to the videos, the virus took around 45 days to spread across the country, whereas the median speed suggests it would have gone from New York to Seattle in under a week. Most grid lines until second 00:19 in the video are black or purple (indicating speeds ≤200 km/day), with high speeds only appearing in the final seconds. This raises doubts about the plausibility of a median speed of ~700 km/day.

Thank you for identifying this error. When investigating this issue, we identified several bugs in the code, which we have now corrected. These were (i) the original approach to generating the hexagonal grid produced hexes of unequal size, and (ii) there was an error in the formula used for calculating speed. We have now corrected the hex grid and speed calculation and updated the results.

In addition, we found that the median speed was frequently zero with the corrected results. This is because, when wave speed is less than one hex width per day, many parts of the wave boundary will not advance in a given day, producing a median speed = 0. As this could be misleading for readers, we now report mean wavefront speed as the summary estimate of speed.

2-Relatedly, the speed values reported in Supplementary Table 5 for wave 2 do not appear to match the wave 2 video. Most estimates in the table are above 500 km/day, with many above 900 km/day (orange in the video legend), whereas the video mostly shows black and purple lines, again suggesting speeds ≤200 km/day. This discrepancy should be addressed.

This issue has now been corrected with the fixes described in the last comment. Thank you for catching this.

3-Are the colours in Figure 3 correct? They seem to be inverted compared to the supplementary figures. If they are correct, then for most of the timeline, wavefront 1 spread faster than wavefront 2, with wavefront 2 only briefly overtaking at the peak. This is the opposite of what is seen for the other thresholds. If the colours are correct, this should be clarified in the limitations section, highlighting the sensitivity of the results to the choice of threshold.

We thank the reviewer for identifying the inverted color scheme in the supplementary plots. We have changed the supplementary plots to ensure a consistent color scheme with **Figure 3**.

4-From a policy perspective, it would be helpful to clarify that the analysis reflects when districts reached the defined threshold—not necessarily when the first case was detected. While this is clear from a methodological standpoint, emphasizing it in more accessible terms would aid broader interpretation.

We have added a sentence to the discussion to address the use of a threshold:

“To produce these estimates, we adopted a definition of the wave front boundary corresponding to >190 infections per 100,000 per day. While this produces a wavefront that will lag behind the first reported case in any given location, it provides a more stable measure of epidemic spread corresponding to meaningful levels of community transmission.” (lines 222-226)

5-The introduction could still benefit from more context about the two waves, including non-pharmaceutical interventions and vaccine rollout status. These contextual details are critical for interpreting the findings, especially for readers less familiar with the field or in future contexts.

We thank the reviewer for the suggestion, which we agree improves the work. We added the following paragraph to the last part of the introduction:

“We note that, in addition to being associated with distinct viral variants, the two waves considered in this study were spreading in conditions that also differed in terms of levels of population immunity (conferred by either previous infections or vaccinations) and the types of non-pharmaceutical interventions being employed. Wave 1 occurred before vaccines were available, and when immunity was acquired for some of the population as a result of infections with the wild-type variants [(21,22)](https://www.zotero.org/google-docs/?wtydZo). Wave 1 also began during a time of nationwide flight mobility restrictions and widespread policies mandating mask use in congregate settings [(5,7,9)](https://www.zotero.org/google-docs/?rN7MU8).

In contrast, wave 2 occurred when there were fewer mobility restrictions and fewer mandatory masking policies in place, though these policies varied markedly by geography within the US. Vaccines had also become available in January 2021 in the US, producing higher levels of apparent population immunity for wave 2 compared to wave 1 [(21,22)](https://www.zotero.org/google-docs/?x4ZTJc). We note wave 2 was largely attributable to the ‘Delta’ variant, which was characterized by its ability to escape immune responses acquired through either previous infection or vaccination.” (line 63–76)

6-The manuscript discusses geographic variation but omits the temporal aspect of spread. Highlighting that the initial spread was geographically limited but temporally faster closer to the peak would strengthen the interpretation of the dynamics.

We thank the reviewer for this suggestion. We have added language to the results to discuss temporal differences within and between waves. To facilitate this discussion, we have divided the waves into three segments, which correspond to the periods between each of the dates presented in **Figures 2** and **4,** and calculated mean speeds for comparison:

“To understand changes in speed over the course of each wave, we calculated mean speed over three time periods: 63-43 days, 42-22 days, and 21-0 days before each wave’s peak. These speed measures increased over the course of each wave, and for each time period, Wave 2 was faster than Wave 1, with mean speeds of 4.4, 13.1, and 30.9 km/day and 2.0, 12.8, and 27.7 km/day, respectively.” (lines 204-208)

7- Figure labels should be revised, as areal wavefront is sometimes mislabelled as speed. Resolution of figures needs to be improved.

We have ensured consistency in the naming of the figure legends throughout the manuscript and have submitted all figures at the highest dpi accepted by the journal submission portal. If accepted, we will work with the journal to ensure high-resolution figures are used for publication.

**Reviewer #2:**

I am satisfied with the author’s revisions and find that the updated document address prior concerns.

We thank the reviewer for their review of the manuscript.

**Reviewer #3:**

Thank you for the thorough revision of my comments. The new version addresses my substantive requests. I have some minor editorial fixes to finalize the manuscript

We thank the reviewer for their review of the manuscript. We have addressed each of the minor editorial issues identified.

Line 102: add space in “μis” → “μ is.”

We have added this space to the manuscript.

Line 103: add space in “ϵ( )are” → “ϵ( ) are.”

We have added this space to the manuscript.

Line 184 (Results, “Wave speed”): fix “overal” → “overall.”

We have fixed this typo.

Line 234 (Discussion): resolve the fragment “adds explanatory factors to the stability observed in diffusion and speed of spread of each wave.” Suggested replacement:
“This adds explanatory factors for the stability observed in diffusion and in the speed of spread of each wave.”

We have resolved the phrase, thank you for the careful reading.

With these small edits, I am satisfied with the revision.

Thank you for identifying these errors!
