# Supplementary material for: Quantifying the spatiotemporal dynamics of the first two epidemic waves of SARS-CoV-2 infections in the United States
Source: PLoS Comput Biol. 2026 Mar 4;22(3):e1013983. doi: 10.1371/journal.pcbi.1013983 (PMC12959703; doi:10.1371/journal.pcbi.1013983)
Supplement: Attachment — Submitted filename: 2026 - Fourth Rebuttal Letter - Waves.docx [file pcbi.1013983.s014.docx]

We thank the editor and reviewers for the opportunity to address comments and revise our manuscript for consideration at PLOS Computational Biology. All line numbers below refer to the lines of the new, clean version of the manuscript. All blue text has been inserted into the manuscript.

**Reviewers' comments:

Reviewer #1:**

Thank you to the authors for addressing the previous comments so thoroughly. I am very pleased with the revisions and the clear improvements in the manuscript. The updated estimates are now much more interpretable and can be confidently used to draw conclusions about virus spread, its drivers, and consequences. I have only three minor final comments.

We thank the reviewer for their patience and attention in reading our manuscript and helping us improve it.

1- I understand the rationale for focusing on the mean given that the median is often zero. However, the median still provides valuable information about the underlying distribution that is not captured by the mean alone. I therefore suggest reporting the median together with the interquartile range (IQR), alongside the mean, at least in the text, to give a more complete picture of the estimates.

We have revised our results to report the mean as the primary outcome, accompanied by the median and interquartile range as secondary outcomes for each estimated value.

2- For Figure 3, please clarify on the y-axis whether the values shown correspond to the mean or the median. In addition, I recommend expanding the legend to more explicitly specify the infection type, time period, and a brief description of the general estimation approach used.

We have edited the figure legend and y-axis labels to reflect that the values are daily means. The figure is reproduced below. We have also added additional context to the figure legend.

“**Figure 3:** Daily mean areal expansion rate and wavefront speed for SARS-CoV-2 infection waves for 63 days preceding the infection per capita peak. (Wave 1: September 8, 2020–November 11, 2020; Wave 2: (July 7, 2021–September 4, 2021). **Top Panel**: Mean daily areal expansion rate. **Bottom Panel**: Mean daily wavefront speed.

Note: Daily areal expansion rate is the change in wave area between day d and d+1. Wavefront speed is the minimal distance from a vertex of the wavefront at day d to a point on the wavefront at day d+1.”

3- While I understand that providing detailed explanations for differences between the two waves is not the primary aim of the study, this discussion would, in my view, substantially enhance the manuscript’s impact. In particular, it would be helpful to include in the discussion some quantitative context regarding population immunity at the time of Wave 2. Based on publicly available data (e.g. estimates suggesting ~50% coverage with two vaccine doses at that time), immunity levels might not have been sufficient to substantially reduce wavefront speed—especially given the immune escape properties of the Delta variant (<https://usafacts.org/visualizations/covid-vaccine-tracker-states/>). I agree with the authors that human mobility likely played the dominant role, which could explain the similar mean speeds of spread across waves (and potentially even more similar medians). However, as currently written, the discussion could be interpreted as suggesting that vaccination and non-pharmaceutical interventions (NPIs) had little to no effect. It is important to emphasize that vaccination uptake and adherence to NPIs were highly heterogeneous across the US. Therefore, the apparent lack of impact on spread velocity may reflect these inequalities in uptake and compliance, rather than the intrinsic effectiveness of these measures. This distinction is important, particularly given strong evidence from other studies that vaccination and NPIs are highly effective when widely and consistently implemented. I appreciate that this was likely not the authors’ intention, but a clarification here would strengthen the interpretation.

We appreciate the reviewer’s concern that our current discussion may not give sufficient weight to the impact of NPIs and vaccination; however, we do want to ensure we do not imply a relationship between these interventions and wave dynamics that we did not investigate. We have added supplemental text to the discussion to add context to the waves:

“While we found that Wave 2 had both a higher maximal speed of wavefront movement and achieved its most significant areal expansion more quickly than Wave 1, the similarity in estimates of wavefront movement speed and the growth rate in wave size for these first two waves is striking. This similarity is remarkable considering the distinct virological, immunological, and policy contexts in which each wave occurred. Wave 1 was due to wildtype SARS-CoV-2 as well as the ‘Alpha’ variant [(37,38)](https://www.zotero.org/google-docs/?rspQwO). Wave 2 was driven by the ‘Delta’ variant (17,18,35) and emerged at a time when approximately 50-60% of the US adult population had received at least one COVID-19 vaccination dose (39, 40). Regional and secular differences in non-pharmaceutical intervention policies, and differences in the levels and types of infection- and vaccine-induced population immunity at the time of these two waves [(17)](https://www.zotero.org/google-docs/?xeL0Nw), make the similarity between the movement patterns of these waves particularly surprising. In a previous analysis, we estimated state-level immunological protection against SARS-CoV-2 infection among US adults. At the onset of Wave 1 in September 2020, we estimated that ~5% of adults were protected against infection. This protection varied minimally between US regions, ranging from ~4% in the Midwest to ~7% in the Northeast. This immunological environment contrasted with that of Wave 2; in July 2021, an estimated ~34% of the US population was protected against SARS-CoV-2 infection, ranging from ~31% in the South to ~38% in the Northeast (41). The consistent speed of expansion of the two waves, each in a different immunological context, may suggest that these expansion rates are relatively stable across immunological contexts. Alternatively, it could be that different factors (population immunity, application of non-pharmaceutical interventions, pathogen characteristics, and underlying patterns of human mobility) had approximately offsetting effects in the two waves we examined [(7,42)](https://www.zotero.org/google-docs/?raLJ3O).”

Additional citations added:

39. Diesel J. COVID-19 Vaccination Coverage Among Adults — United States, December 14, 2020–May 22, 2021. MMWR Morb Mortal Wkly Rep [Internet]. 2021 [cited 2026 Jan 23];70. Available from: https://www.cdc.gov/mmwr/volumes/70/wr/mm7025e1.htm

40. Datar RS, Fette LM, Hinkelman AN, Hammershaimb EA, Friedman-Klabanoff DJ, Mongraw-Chaffin M, et al. Factors associated with COVID-19 vaccination during June–October 2021: A multi-site prospective study. Vaccine [Internet]. 2023 May 11 [cited 2026 Jan 22];41(20):3204–14. Available from: https://pmc.ncbi.nlm.nih.gov/articles/PMC10063571/

41. Klaassen F, Swartwood NA, Chitwood MH, Lopes R, Haraguchi M, Salomon JA, et al. National- and State-level SARS-CoV-2 Immunity Trends From January 2020 to December 2023: a Mathematical Modeling Analysis. J Infect Dis [Internet]. 2025 Oct 23 [cited 2026 Jan 23];jiaf532. Available from: <https://doi.org/10.1093/infdis/jiaf532>

[42.](https://www.zotero.org/google-docs/?EcjNVr)	[Firestone MJ. COVID-19 Outbreak Associated with a 10-Day Motorcycle Rally in a Neighboring State — Minnesota, August–September 2020. MMWR Morb Mortal Wkly Rep [Internet]. 2020 [cited 2024 May 21];69. Available from: https://www.cdc.gov/mmwr/volumes/69/wr/mm6947e1.htm](https://www.zotero.org/google-docs/?EcjNVr)

4- Please check references. Is reference 40 correct? I was expecting a publication about immunity in the US but it is instead about B.1.1.7 in the UK.

We thank the reviewer for their careful review of the reference list. We have double-checked all the citations to ensure their correctness. The citation mentioned above should be:

“Earnest R, Uddin R, Matluk N, Renzette N, Turbett SE, Siddle KJ, Loreth C, Adams G, Tomkins-Tinch CH, Petrone ME, Rothman JE. Comparative transmissibility of SARS-CoV-2 variants delta and alpha in New England, USA. Cell Reports Medicine. 2022 Apr 19;3(4).”
